# Supplementary material for: Recruiting on the Spot: A Biodegradable Formulation for Lacewings to Trigger Biological Control of Aphids
Source: Insects. 2019 Jan 5;10(1):6. doi: 10.3390/insects10010006 (PMC6358976; doi:10.3390/insects10010006)
Supplement: Supplementary file 1 [file insects-10-00006-s001.pdf]

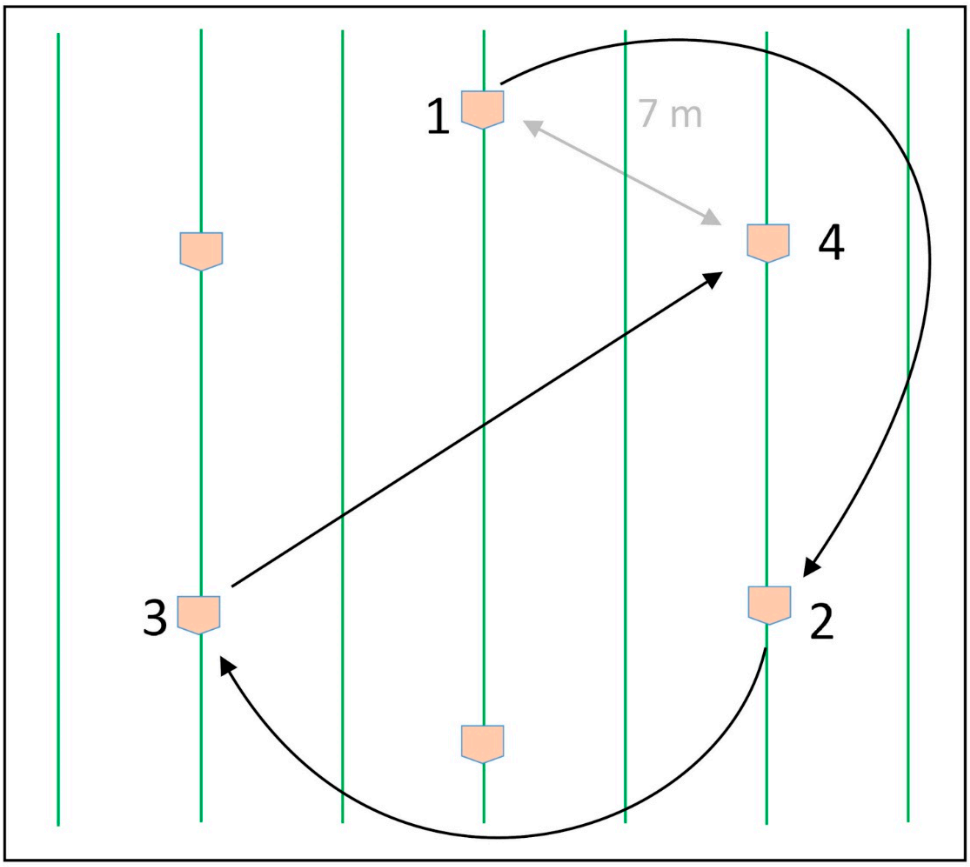

Figure S1. Schematic representation of the trapping experiment with McPhail traps in apple orchards and how they were rotated. Vertical lines represent apple rows. Arrows represent how every single trap was moved to new positions over the 4-week period.

Table S1. Coefficient estimation for volatile release.

Methyl Salicylate	Phenylacetaldehyde	Acetic Acid	2-Heptenal	2-Phenylethanol	eBenzaldehyde	Benzyl Acetate	Benzyl Alcohol	Hexanoic Acid
5,760,187,599	3,587,174,895	38,815	31,355,843	2592	44,116	7566	19,646	3.09E-12
−1,926,545,605	6,223,99,348	6365	−(*)	2113	−4965	−4550	−2778	−(*)
−53,682,997	-271,822,842	28,915	−14,414,900	520	4691	1445	8618	3907
335,604,420	-301,093,871	12,768	−23,427,108	1018	9111	2524	8051	1830
67,134,466	-508,537,215	−5573	−29,826,412	2081	11,104	3029	8155	431
−1,963,516,800	−1,147,613,144	−13,588	31,355,843	3484	8671	4108	7229	3.09E-12
−1,588,105,844	−1,827,998,546	−1071	−(*)	−2214	−8554	−3395	−9234	−(*)
−2,609,670,583	−2,917,682,029	−2567	−(*)	−4308	−21,152	5048	−12,104	−(*)
−2,838,940,784	−3,258,361,819	2153	−(*)	−6250	−27,272	−5256	−16,428	−(*)
−1,644,052,131	−2,988,111,653	−744	−(*)	−8190	−35,274	−7124	−19,963	−(*)
